# Influence of Endodontic Cavity Design on Interfacial Voids, Class II Resin Composites Sealing Ability and Tooth Fracture Resistance: An In Vitro Study

**DOI:** 10.3390/jcm13196024

**Published:** 2024-10-09

**Authors:** Abdurrahman S. Assalman, Faisal Al Onaizan, Moataz Elgezawi, Khalid S. Almulhim, Moamen A. Abdallah, Dalia Kaisarly

**Affiliations:** 1 Department Restorative Dental Science, College of Dentistry, Imam Abdulrahman Bin Faisal University, Dammam 34212, Saudi Arabia; falonaizan@iau.edu.sa (F.A.O.); malgizawi@iau.edu.sa (M.E.); ksalmulhim@iau.edu.sa (K.S.A.); 2 Department of Substitutive Dental Science, College of Dentistry, Imam Abdulrahman Bin Faisal University, Dammam 34212, Saudi Arabia; maabdalla@iau.edu.sa; 3 Department of Conservative Dentistry and Periodontology, University Hospital, LMU Munich, 80336 Munich, Germany

**Keywords:** contracted endodontic access, obturation voids, class II restoration, cervical sealing, micro-CT, fracture resistance

## Abstract

**Objective**: The aim of this in vitro investigation is to study the effect of endodontic cavity design on interfacial voids, class II resin composite sealing ability, and fracture resistance in mandibular premolars. **Methods**: A total of 48 single-rooted mandibular premolars received compound class II preparations with either traditional flare access cavities (group A) or contracted endodontic cavity preparations (group B). Each study group was subdivided according to the coronal restoration into two sub-groups as α and β. In the α group, a microhybrid composite was used after etch-and-rinse bonding technique using an MDP-containing universal adhesive. In the β group, a self-adhesive composite was used as coronal restoration after endodontic treatment (*n* = 12) for each subgroup. A micro-CT analysis was performed to assess the obturation interfacial voids and tracing of class II cervical interfacial adaptation. The tooth fracture resistance testing was then performed adding an extra group of 12 sound non-prepared teeth, which were tested as the control for fracture strength testing. A one-way ANOVA and post-hoc testing were used together with descriptive statistics for an analysis of the mean values of obturation interfacial voids. A two-way ANOVA was used to assess the fracture resistance test results, and to find the influence of endodontic access design and the type of composite material on the fracture resistance testing. Chi-square testing was employed to analyze the cervical interfacial seal of the class II restorations. **Results**: A one-way ANOVA revealed that there were no statistically significant differences between test groups in the amount of obturation interfacial voids (*p* > 0.05). There were no statistically significant differences between test groups in terms of cervical interfacial sealing ability (*p* > 0.05). A two-way ANOVA revealed that no statistically significant differences between test groups including the control group existed in terms of the fracture resistance testing (*p* > 0.05). **Conclusions**: Although it does not improve tooth fracture resistance, the contracted endodontic access cavity does not deteriorate the quality of obturation in terms of the interfacial porosity. The self-adhesive composite does not improve the efficiency of cervical interfacial adaptation or tooth fracture resistance regardless of the endodontic access cavity shape, yet it revealed a substantial load-bearing capacity.

## 1. Introduction

The ultimate objective of an optimum and long-term successful root canal treatment is the virtual elimination of infection in the root canal system, the treatment of apical periodontitis, and the confirmation of tight obturation with a three-dimensional hermetic seal cervically and apically to prevent re-infection [1].

Each individual tooth has anatomical characteristics that seem to have an impact on the endodontic treatment outcome and long-term prognosis. It has been demonstrated that following endodontic treatment, roots with narrower mesiodistal dimensions than buccolingual dimensions, like in mandibular premolars, are more susceptible to fracture. Besides, different root canal instruments’ material of construction and kinematics, amount of dentin removal, and stresses induced due to instrumentation on canal walls have evident influences on the root canal dentin wall cracking, crack propagation potentials, and, hence, on the root fracture strength [2,3]. The literature shows that when compared to mandibular canines, instrumented mandibular premolars have higher risk of fracture than un-instrumented premolars [4]. Restoring the mandibular premolars is challenging when optimum biomechanical integration is the objective [5]. Mandibular premolars have been considered as an enigma to the endodontist and present challenges in endodontic treatment with high incidence of failure and flare ups. The lingual inclination of the crowns of mandibular premolars, narrow mesiodistal dimensions, and variations in root canal system morphology can interfere with suitable visibility, optimum canal system instrumentation, and irrigation, which necessitate adequate attention during endodontic cavity preparation. The challenges in root canal system morphology are true even in single-rooted mandibular premolars with one canal and single apical foramen that show cross-sectional shape variation along the course of the root canal. This can increase the incidence of preparation errors and shape aberration [6,7,8,9,10].

Endodontic instruments and instrumentation have been reported to significantly contribute to the overall strength of the remaining tooth structure, the quality of postinstrumentation root canal shape, and configuration, as well as to the quality of obturation [5,11]. The contracted endodontic cavity has received an increasing interest for its conservation of tooth structure. A 3D finite element analysis supported a proposed improvement in the mechanical resistance of teeth during function with minimal invasive access cavities in comparison to the conventional access cavities [12]. On the contrary, a recent report has indicated that traditional endodontic cavities preserve the original root canal anatomy better than contracted endodontic cavities in lower molar teeth [13].While another investigation spotted that conservative access cavities demonstrated an inferior sealing quality and a greater incidence of obturation voids than traditional cavities [1]. Currently there is inadequate evidence to support the expanded clinical implementation of contracted endodontic access cavities or the training of undergraduate and postgraduate students in different educational programs [14,15,16].

The cervical margins of class II resin composites are challenging clinical locations with respect to the effectiveness of the bonding to the tooth’s structure, the marginal and interfacial seals, the long-term degradation resistance, and the clinical reliability [17]. There is a continuous turnover and modification of composite resin types. Among the recently emerging resin composites is the self-adhesive composite that aims to simplify resin composite restoration procedures and diminish technique sensitivity [18].

Deep subgingivally located gingival margins constitute an additional clinical challenge when tight marginal seals and tooth fracture resistance are of concern [19,20]. To bring the deep subgingivally located proximal gingival margins to supragingival locations for an optimum resin composite restoration, a base of direct resin composite has been used in the so-called deep marginal elevation (DME) [21]. In vitro testing of the efficiency of restorative materials and restoration techniques, including the fracture resistance of endodontically treated teeth, quality of obturation, and coronal restorations sealing ability, can be instrumental in reliability assessments [16,22,23,24].

Therefore, the aim of this in vitro study was to assess the obturation interfacial voids and the fracture resistance of endodontically treated mandibular premolars prepared by contracted and conventional endodontic access with class II cavity preparation. Moreover, the cervical interfacial sealing ability of class II self-adhesive versus the conventional resin composite will be investigated. The alternative hypotheses were that the contracted endodontic access cavity does not increase the obturation interfacial voids or tooth fracture resistance and that the self-adhesive composite does not improve the cervical interfacial adaptation or tooth fracture resistance.

## 2. Material and Methods

The study was conducted at the College of Dentistry at Imam Abdulrahman bin Faisal University. An ethical approval of the institutional review board was attained (IRB-PGS-2024-02-052) and approval dated 21 January 2024, adhering rigorously to all protocols and ethical principles outlined in the Helsinki Declaration (1964) and its subsequent revisions.

### 2.1. Sample Size Calculation

Assuming mean (±SD) fracture strengths, respectively, 1616.3 ± 106.53 and 1369.9 ± 253.9 of the control group versus the traditional endodontic cavity (TEC) group Saberi et al., 2020 [22], µ1 = 1616.3, µ1 = 1369.9, and σ = 180.2, on 5% level of significance, 95% confidence interval, and 85% power of test, the minimum target sample size per group was 10 specimens. A sample size of 10 specimens per each study group was used previously in similar studies [22,25].

### 2.2. Samples Collection and Allocation

Sixty human single-rooted mandibular premolars that were recently extracted due to orthodontic reasons were collected for the purpose of this study following the attained ethical approval policies. Each tooth was assessed using a CBCT scan; a Kodak 9000 3D (Carestream Health, Inc., Marnela-Vallee, France) was employed for the acquisition of images of each individual tooth, utilizing a spatial resolution of 200 µm [26]. Specifically, teeth with not only one root canal and a fully formed root apex without any defects or cracks on the surface but also have restorations with a root curvature of 0–20° (according to Schneider [27]) and with similar sizes and crown dimensions were assigned for the study. The collected teeth were properly cleansed of blood stains using ultrasonic cleansers and were then immersed in a 0.02% sodium azide solution at room temperature.

Group allocations and grouping were as follows: teeth were randomly allocated to the following main groups: Group (A) traditionally flared access with two subgroups (α and β), each with *n* = 12 (A α group as positive control); Group (B) contracted endodontic cavity preparation with two subgroups (α and β), each with *n* = 12; Group (C) the control group (negative control) with (*n* = 12) intact mandibular single-rooted premolars with no endodontic treatment or restorative procedures. For the subgroups (α and β) in both Group A and Group B: α subgroups were restored with a microhybrid composite after total-etch technique using an MDP-containing universal adhesive, while β subgroups were restored with a self-adhesive composite. In the 48 teeth within the test groups, compound class II occluso-proximal cavities were prepared with standardized dimensions.

### 2.3. Class II Cavity Preparation

Specimens received compound class II cavities with standardized dimensions as follows: a buccolingual width of 1/3 of the intercuspal distance, buccal and lingual walls parallel to each other and to the long axis of the tooth, a 1.5 mm thick flat gingival seat, and a proximal gingival margin located 1.5 mm below the cementoenamel junction and butt joint margins were prepared in each tooth. The standardization of preparations was inspected by the repeated periodontal probe measurements during cutting procedures. Cavities were prepared using a high-speed hand piece under profuse air–water coolant. The preparation started by gaining access through the enamel occlusally using a round diamond stone and completed with a parallel sided carbide fissure bur.

### 2.4. Access Cavity and Radicular Preperation

#### 2.4.1. Group A: Traditional Access Cavity

Traditional flare access cavities were employed. The No. 1 round carbide and Endo Z burs (Dentsply Sirona International, York, PA, USA) were used for the traditional occlusal flaring endodontic access cavities.

#### 2.4.2. Group B: Contracted Access Cavity

A No. 1 round carbide bur (Dentsply Sirona International, York, PA, USA) was used for the contracted occlusal endodontic access cavities without occlusal flaring.

#### 2.4.3. Cleaning, Shaping, and Obturation of the Root Canal

After the access cavity preparation, a working length was taken by a K-file size 10 of 0.5 to 1 mm from the root’s apex, then cleaning and shaping were standardized with the use of a Vortex rotary system with the crown-down technique employing a brushing motion up to size 35, 0.04 and 10 mL of 5.25% sodium hypochlorite with a 30-gauge side vented needle alternatively.

Paper points were used to dry the canal, which was then obturated with gutta-percha with the warm vertical compaction technique (Fast-Pack Pro and Fast Fill, Eighteeth, Changzhou Sifary Medical Technology Co., Changzhou, China) and using AH Plus Root Canal Sealer (Maillefer Instruments Holding, Ballaigues, Switzerland).

### 2.5. Coronal Restoration

A Myler strip tightened to a Toffelmire matrix retainer was applied to the tooth before the restorative procedure.

#### 2.5.1. Microhybrid Resin Composite (Filtek Z250) (α Groups)

The total-etch technique was employed as an adhesive approach. A 34% phosphoric acid (Scotchbond Universal Etchant, 3M ESPE St Poul, MN, USA 3M) was used to etch the prepared cavity walls for 15 s. This was followed by washing with water for 10 s and gently air drying for 3 s. An MDP-containing universal adhesive (3M™ Single Bond Universal Adhesive, 3M) was then applied with a micro-sponge, rubbed for 10 s, then gently air dried for 5 s. This was followed by light curing of the adhesive for 20 s once occlusally and proximally against the preparation using an LED light-curing unit (MiniLED Standard, Acteon, Merignac, France) that provides a power output of 925 mW/cm^2^ to 1250 mW/cm^2^. The resin composite was then inserted in successive horizonal increments of 2 mm each until the complete filling of the cavity (Filtek Z 250 microhybrid composite, 3M ESPE ST Poul USA) with the consequent light curing of each increment for 40 s occlusally. Before occlusal light curing the last increment, a plastic instrument was used to initially provide the occlusal anatomical features. An additional 40 s of proximal light curing was performed after occlusal curing of the last increment. The matrix was then removed, followed by the removal of proximal marginal flashes using a surgical lancet. A carbide bur (12 bladed football, Diatech, Pleasant, SC, USA) was used, followed by white stone (Ammdent, FD-DD001, Ajitgarh, Punjab, India) for the finishing of the restoration [28].

#### 2.5.2. Self-Adhesive Composite (β Groups)

The prepared cavities were rinsed using a water spray, then air dried for 5 s. The self-adhesive composite (Vertise Flow, Kerr, CA, USA) was then inserted into the prepared coronal cavity and light cured in the following manner: initial layer of 0.5 was applied over the prepared cavity floor and walls, then brushed vigorously for about 15 s before light curing once occlusally and once proximally for 20 s each direction. This was followed by the successive injection of the composite until completely filling the cavity. Before light curing, the excess composite was removed with a plastic instrument to initially provide the anatomical features of the tooth. The matrix was then removed, followed by the removal of the marginal flashes using a surgical lancet. A finishing was then executed similar to the previous group [29].

### 2.6. Dye Penetration Test

A micro-CT analysis was used employing a silver nitrate dye immersion for the assessment of the cervical interfacial sealing ability of the composite restoration at the proximal gingival interface [30]. Upon finishing the coronal restoration, teeth were painted with two layers of clear nail varnish except 1 mm above and 1 mm below the gingival margins. The specimens were then immersed in a 50% aqueous solution of silver nitrate under dark conditions overnight at room temperature. The specimens were then removed from the dye solution and rinsed with water for 1-min.

For each respective specimen, silver nitrate penetration was then assessed through Micro-CT scanning. The dye penetration was scored according to the following categories:

0 = No penetration of silver nitrate along the gingival tooth-restoration interface.

1 = Silver nitrate reaches up to 1/2 of the gingival seat.

2 = Silver nitrate reaches up to more than 1/2 of the gingival seat.

3 = Silver nitrate extends beyond the axial wall.

4 = Silver nitrate reaches the root canal system.

### 2.7. Micro CT Scan

**Figure 1:**
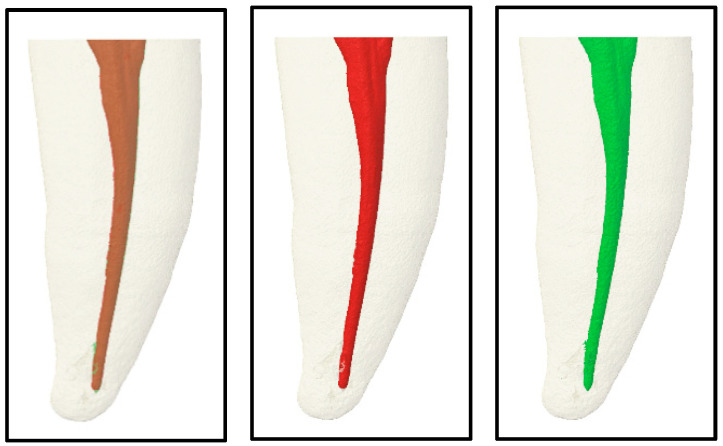
3D Micro-CT image for root canal system. After root canal instrumentation (green), After obturation (red), and Superimposed image (brown) of two scans show percentage of obturation voids between two scans.

Micro-CT scans at College of Dentistry, Imam Abdulrahman Bin Faisal University lab, were used for the assessment of obturation interfacial voids and the interfacial sealing ability (the extent of silver nitrate penetration). The teeth were embedded with the root portion in clear resin measuring 15 * 15 mm. The Skyscan 1172 scanner Bruker Corp.,Kontich, Belgium, was employed for imaging, utilizing a source voltage of 100 kV, a source current of 100 uA, an Al + Cu filter, an exposure time of 4700 ms, a rotation step of 0.5 degrees, and a random movement of 10 units during the 360° rotation. A reconstruction was performed using the NRecon version 1.7.3.0, Bruker, Kontich, Belgium software program, with specific parameters including a smoothing level of 6, a ring artifact correction level of 6, and a beam hardening correction of 20. Two scans per each study sample were undertaken: the first scan focused solely on the roots following the root canal preparation, while the second scan encompassed the crown and root after the root canal treatment, the tooth restoration, and the dye immersion (oversized scan). 3D software analysis and superimposition were executed by SkyScanCT-Volume v2.2. An analysis of interfacial voids’ sizes was assessed by superimposition of the two scans, then a calculation of voids’ volume in mm^3^ [31]. Figure 1: 3D Micro-CT image for root canal system.

**Figure 2:**
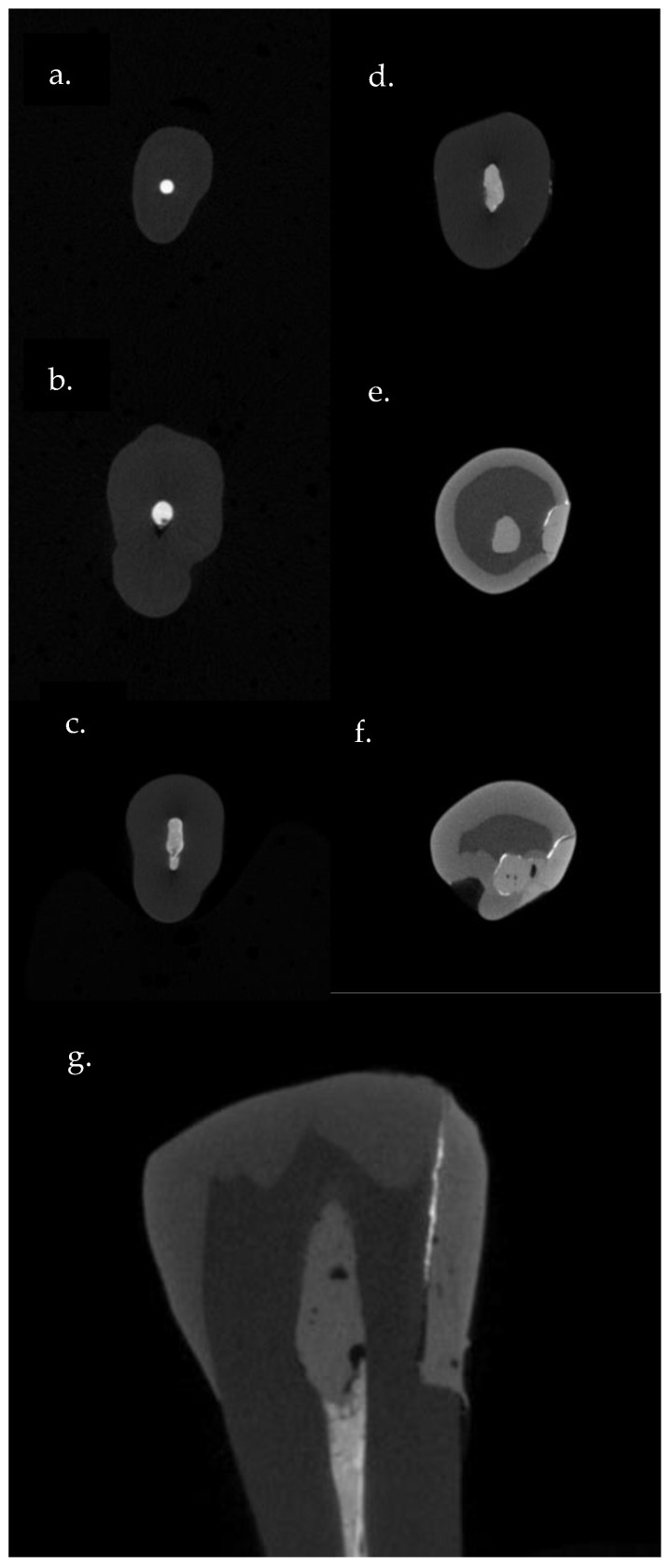
Micro-CT scan sections of representative sample: (**a**) apical section, (**b**,**c**) middle-third section, (**d**) coronal-third section, (**e**,**f**) axial sections of tooth crown show penetration of silver nitrate underneath the composite material, and (**g**) sagittal section of tooth crown shows penetration of silver nitrate.

For the interfacial sealing ability (extent of silver nitrate penetration), only crown sections (axial and sagittal) of each sample were used for the determination of silver nitrate penetration (Figure 2).

### 2.8. Fracture Resistance Test

**Figure 3:**
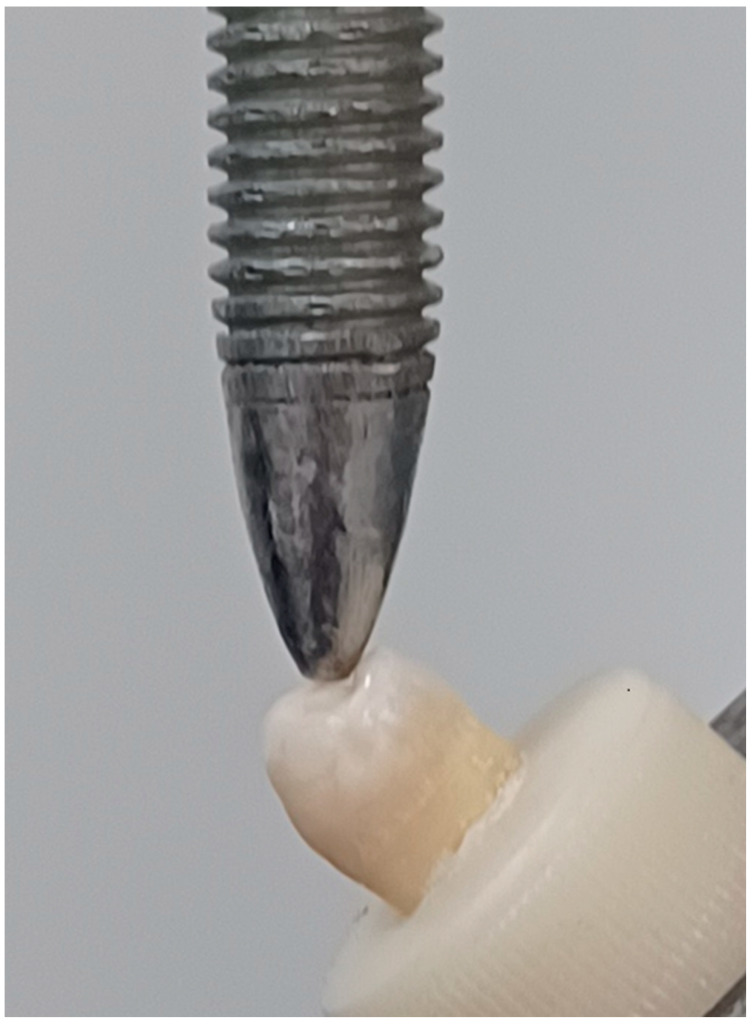
Fracture resistance testing in a universal testing machine, sample mounted at 45°.

Each sample was fixed in a universal testing machine (WDW-100, Changchun, China), with a stainless-steel sphere (5 mm in diameter) contacting the lingual plane of the buccal cusps. Then a load was applied at a crosshead speed of 1.0 mm/min and at an angle of 45° to the long axis of the tooth until fracture occurred [32], Figure 3. The fracture resistance values in newton were recorded and tabulated.

### 2.9. Statistical Analysis

The obturation interfacial voids’ results were assessed using a one-way ANOVA. The fracture resistance test results were assessed using a two-way ANOVA to show the influence of endodontic access design and the type of composite material on the fracture resistance test. The Tukey test was used in both ANOVAs for multiple comparisons between the groups. A chi-square test was used to assess penetration of silver nitrate data. All the analyses were performed using SPSS software (IBM, Armonk, NY, USA, SPSS Statistics 29.0.2.0), with a probability level set to 95% (*p* < 0.05).

## 3. Results

### 3.1. Obturation Interfacial Voids

**Figure 4:**
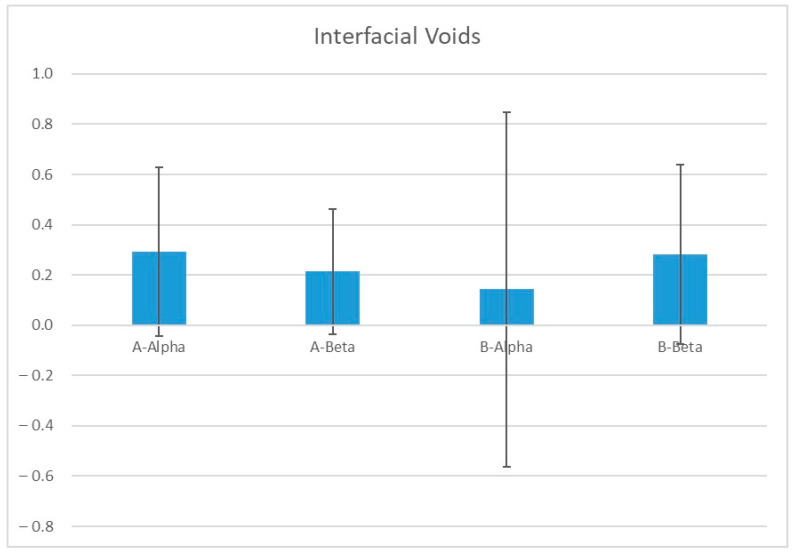
Obturation interfacial voids, mean, and standard deviation for all groups. A: traditional access cavity, B: contracted access cavity, Alpha: microhybrid composite, and Beta: self-adhesive composite.

Although the total volume of the obturation interfacial voids’ mean (SD) was variable as it was highest in A-Alpha, 0.29 mm^3^ (±0.34), and lowest in B-Alpha, 0.14 mm^3^ (±0.70), the overall mean of A groups was 0.25 mm^3^ (±0.30) and the overall mean of the B groups was 0.21 mm^3^ (±0.56), which bears similar interfacial void volume. A one-way ANOVA analysis in all groups revealed no statistically significant differences in mean between the groups (*p* > 0.05). Figure 4 Obturation interfacial voids, means, and standard deviation for all groups & Table 1: Obturation interfacial voids, significance levels, and confidence intervals for group comparisons.

### 3.2. Cervical Interfacial Sealing Ability

Most of the restored teeth within each group revealed no penetration—the least penetration in the micohybrid composite group (A-Alpha and B-Alpha), while the self-adhesive flowable composite groups exhibited more prevalence of penetration. The percentage of no penetration of silver nitrate was highest in B-Alpha (100%) and lowest in B-Beta (58.3%). A chi-square test for the cervical interfacial sealing ability of the composite material revealed no statistically significant differences between all test groups (*p* > 0.05) (Table 2).

### 3.3. Tooth Fracture Resistance

**Figure 5:**
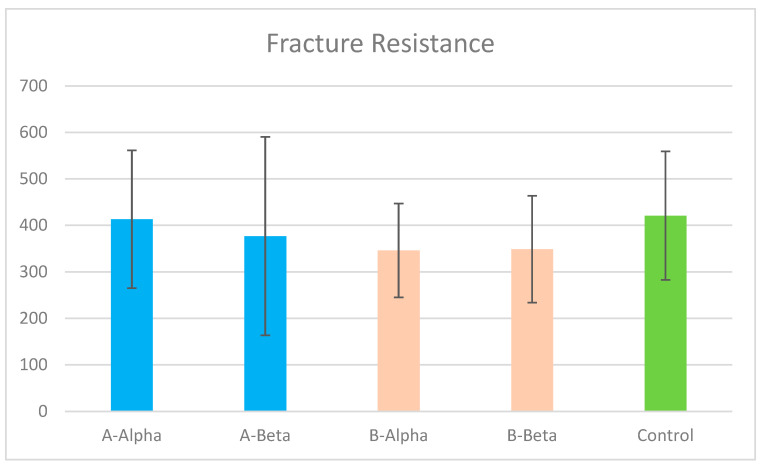
Fracture resistance results, mean and standard deviation. A: traditional access cavity, B: contracted access cavity, Alpha: microhybrid composite, and Beta: self-adhesive composite.

Overall and within the two endodontic access designs, etch/rinse and microhybrid composite groups revealed slight increases in fracture resistance compared to the self-adhesive flowable composite groups. The mean (SD) of fracture resistance was highest in A-Alpha, 413.26 (±148.07), and lowest in B-Alpha, 346.10 (±101.11), as the contracted access design demonstrated slight decreases in fracture resistance when compared to the traditional access cavity. The results of the fracture resistance test show no statistically significant differences in means between all groups including the control group (*p* > 0.05). The control group of non-prepared teeth had the greatest fracture resistance, significantly more than all other test groups, 420.94 (±138.28) (*p* > 0.05). Figure 5: Fracture resistance results, mean and standard deviation. & Table 3: Fracture resistance results, significance levels, and confidence intervals for group comparisons. The Spearman correlation shows a positive correlation between fracture resistance and the cervical interfacial sealing ability results rho (0.266), but there were no statistically significant differences between them (*p* > 0.05). Table 4: Spearman correlation test between fracture resistance test and cervical interfacial sealing ability, and significant level.

## 4. Discussion

The turn-up of minimal endodontic access cavities has always been derived by the need to conserve tooth structure while penetrating the roof of the pulp chamber as a presumable way for improving tooth fracture resistance. However, concerns remained regarding the possibility of the subsequent inadequacies of the instrumentation and filling of the root canal system, iatrogenicities in the form of missing canals and separated instruments, left-behind necrotic tissue, bacterial nidi, or discoloring restorations [16].

The presence of inherent diversity in teeth, particularly in the anatomy of the root canal system and pulp chamber, is a crucial factor in the success of endodontic treatment and an influencing factor that can introduce bias in the comparative research [6,7].

In our study, various measures were taken to ensure that the samples could be compared effectively. These involved conducting a preliminary screening of the teeth based on their anatomical and morphological characteristics, such as length, volume, and surface area, using CBCT scanning and assigning only mandibular premolars of similar size, shape, pulp, and root canal anatomical features, as per the study’s design [1].

The Vortex system was used as it has constant tapering, which gives a standardized tapering of the root canal [33]. Future investigations should include various rotary systems for assessing the effectiveness of conservative access cavities to draw more extensive conclusions.

Previous studies have indicated that the warm vertical compaction obturation technique is more advantageous than the lateral condensation technique in less extensive and more conservative access cavity designs [16,34]. Therefore, to avoid the inevitable variability in the lateral condensation technique, only the warm vertical compaction obturation technique was used in this study.

This study followed previous investigations by immersing the specimens in silver nitrate for 24 h before the micro-CT tracing of the cervical interfacial dye penetration [30,35,36].

Our results that the contracted and conventional access cavities produce similar interfacial obturation voids suggest accepting the provided alternative hypothesis, which proposes the contracted access cavity does not increase the obturation interfacial voids. A potential explanation of these findings might be the use of the warm compaction technique for obturation that requires less access and can be more suitable for more conservative access cavities [16,34]. Moreover, our findings that no statistically significant differences in the fracture resistance of mandibular premolars regardless of the endodontic access cavity, conventional or contracted, support accepting the alternative hypothesis that the contracted access cavity does not improve tooth fracture resistance. This is in line with previous similar studies [31,37,38] that the contracted access cavity does not improve tooth fracture resistance. On the other hand, other investigations reported the variable fracture resistance improvements with more conservative endodontic access cavities [12,39,40]. Nevertheless, a recent systematic review, a meta-analysis, and a decision-making protocol [41] have concluded that more conservative access cavities are not inevitably, biologically, and mechanically advantageous, particularly in teeth with one or more missing surfaces, which match our study’s design, where the class II compound was prepared before the endodontic access cavity preparation.

Our findings that using a self-adhesive composite does not improve the cervical interfacial seal lead to an acceptance of the respective alternative hypothesis. These findings might contradict older reports indicating that the self-adhesive flowable composite Vertise flow provides a better seal when used as a liner in class II restorations with gingival margins below the cementoenamel junction. However, in the current study, the self-adhesive composite was used to entirely fill the coronal cavity. A self-adhesive composite has been also reported to have a satisfactory clinical performance and easier application [42,43,44,45], which do not apply to our study’s design or testing methodology.

Moreover, the present study’s observations that a self-adhesive flowable composite does not improve tooth fracture resistance suggest acceptance of the alternative hypothesis. This is in agreement with the previous findings of Atalay et al. [46], who used a bulk-fill flowable composite versus other resin composites to restore MOD cavities in endodontically treated teeth without any improvement in fracture resistance.

The Vertise Flow self-adhesive composite [29] used in the β group is a flowable composite with a greater organic matrix content and less filler content than the Filtek Z250 microhybrid composite used in the α group of the current study. Our findings that there are no significant differences between α and β groups in terms of the interfacial sealing ability and tooth fracture resistance might be related to the small occlusoproximal cavity size in the used mandibular premolar teeth with little variability in volume between restorations. Although a flowable composite can show more elastic behavior during polymerization shrinkage with less tendency of interfacial separation, the material has a greater organic matrix than a microhybrid composite with a greater net contraction [47,48]. The same explanation might elucidate the similar fracture resistance results between the two groups and the control group of unprepared teeth. The similarity of fracture resistance between the study groups probably suggests the validity of the Vertise Flow self-adhesive composite in restoring endodontic access with class II cavities. Succeeding studies using lager sizes and class II coronal restorations of endodontically treated teeth are needed to draw a broader conclusion.

In the present study, the self-adhesive Vertise Flow composite was used for the reported advantages of reduced time, steps of application, and, hence, a decreased technique sensitivity and to make use of greater flexibility of the material when compared to the microhybrid composite. These merits appear to be advantageous, particularly at the deep proximal marginal location restorations used in this study.

The overall investigation of coronal and radicular restorations’ protocol in endodontically treated teeth could present variable outcomes based on the study’s design. In a previous study of a different design, Tekçe et al. investigated the obturation voids in composite restorations and fracture resistance in mandibular premolars using only conventional endodontic access cavities with lateral compaction. They found similar tooth fracture resistance in the tested composite groups [49]. In our study, the root canal obturation voids, tooth fracture resistance, and cervical interfacial adaptation were tested comparing the conventional access cavities to the contracted access cavities. The fracture resistance outcome considering the composite types revealed a comparable load-bearing capacity of the self-adhesive composite.

Boscatto et al. studied mandibular premolars and the percentage of hard tissue removal and fracture resistance of teeth using the conventional and conservative access cavities with a coronal composite versus a composite and fiber-post restorations. They found similar tooth fracture resistance favoring minimal intervention procedures [50].

In a systematic review and meta-analysis, Zarow et al. [51] investigated the fracture resistance of endodontically treated teeth with and without a composite build-up material, and reported that restoring endodontically treated teeth with a higher filler resin composite increases tooth fracture resistance compared to conventional composites.

In a distinct difference, our study demonstrated that, regardless of the coronal restoration used, the flowable self-adhesive, a conventional microhybrid-inserted following total-etch bonding approach, and contracted access cavities do not improve fracture resistance values, which is in agreement with previous studies [26,52,53]. Further investigations are still needed to validate the clinical reliability of the contracted access cavity.

Our experience while restoring the current study samples confirms the previously reported drawbacks of contracted access cavities since there was a clear difficulty in removing traces of the root canal fillings’ materials from the pulp chamber before the placement of the coronal restoration, despite utilizing ultrasonic tips, which led to the magnification’s increased time consumption to finalize the procedure [54,55].

The current study has a number of limitations due to its in vitro nature and the respective descriptions of fracture resistance and dye penetration tests that do not simulate the clinical conditions. The current study was principally designed to investigate the immediate post-restoration influences of the contracted versus conventional access cavities, as well as the self-etch flowable versus conventional microhybrid composite. Although the long-term effectiveness and longevity of the studied restorations might significantly affect the reliability of the test restorative techniques and materials, they are still beyond the scope of this study. The high standard deviation presented in the results indicates the data are scattered into a wide variety of values around the mean. Such intragroup variability might be related to unavoidable variation in teeth sizes, root canal systems’ details in shapes, sizes, and course, as well as differences in the teeth’s structural composition and qualities. Future in vitro studies are required for more reliable outcomes.

Future studies should investigate the long-term reliability of the test cavities and restorations, and consider all clinical influences of thermal, pH, and load cycling, as well as other clinical environmental factors of bacterial activities, humidity, and consequently progressive time-dependent biodegradation. More NiTi rotary systems, obturation techniques and materials, and different coronal restorations should be incorporated into future studies’ designs to be able to drive wide-range conclusions. Successive studies should consider in greater depth the effectiveness of instrumentation and obturation when the contracted and other conservative endodontic access cavities are prepared before implementing them in educational programs of endodontics and comprehensive clinical dentistry courses [56].

## 5. Conclusions

Under the circumstances of the current investigation and putting into consideration all study limitations, the following conclusions could be drawn: the contracted endodontic access cavity does not provide a mechanical advantage by improving tooth fracture resistance and has no negative influence on the obturation interfacial porosity. A self-adhesive composite does not improve the cervical interfacial adaptation or tooth fracture resistance regardless of the endodontic access cavity design.

## Figures and Tables

**Table 1:** Obturation interfacial voids, significance levels, and confidence intervals for group comparisons.

(I) Group	(J) Group	Sig. (*p*-Value)	95% Confidence Interval	
			Lower Bound	Upper Bound
A-Alpha	A-Beta	0.97	−0.41	0.57
	B-Alpha	0.85	−0.34	0.64
	B-Beta	1.00	−0.48	0.50
A-Beta	A-Alpha	0.97	−0.57	0.41
	B-Alpha	0.98	−0.42	0.56
	B-Beta	0.98	−0.56	0.42
B-Alpha	A-Alpha	0.85	−0.64	0.34
	A-Beta	0.98	−0.56	0.42
	B-Beta	0.87	−0.63	0.35
B-Beta	A-Alpha	1.00	−0.50	0.48
	A-Beta	0.98	−0.42	0.56
	B-Alpha	0.87	−0.35	0.63

A: traditional access cavity, B: contracted access cavity, Alpha: microhybrid composite, and Beta: self-adhesive composite.

**Table 2:** Cervical interfacial sealing ability (distribution of silver nitrate penetration) outcomes across experimental groups.

			No Penetration	Silver Nitrate Reaches up to 1/2 of Gingival Seat	Silver Nitrate Reaches up to More than 1/2 of Gingival Seat	Silver Nitrate Extends beyond the Axial wall	Silver Nitrate Reach up to Root Canal System	Total
Group	A-Alpha	Count	10	1	0	0	1	12
		% within Group	83.3%	8.3%	0%	0%	8.3%	100%
	A-Beta	Count	8	2	1	1	0	12
		% within Group	66.7%	16.7%	8.3%	8.3%	0%	100%
	B-Alpha	Count	12	0	0	0	0	12
		% within Group	100%	0%	0%	0%	0%	100%
	B-Beta	Count	7	1	4	0	0	12
		% within Group	58.3%	8.3%	33.3%	0%	0%	100%
Total		Count	37	4	5	1	1	48
		% within Group	77.1%	8.3%	10.4%	2.1%	2.1%	100%
Pearson Chi-Square (*p*-value)			0.110					

A: traditional access cavity, B: contracted access cavity, Alpha: microhybrid composite, and Beta: self-adhesive composite.

**Table 3:** Fracture resistance results, significance levels, and confidence intervals for group comparisons.

(I) Group	(J) Group	Sig. (*p*-Value)	95% Confidence Interval	
			Lower Bound	Upper Bound
A-Alpha	A-Beta	0.97	−134.34	207.13
	B-Alpha	0.80	−103.58	237.89
	B-Beta	0.83	−106.46	235.00
	Control	1.00	−178.41	163.05
A-Beta	A-Alpha	0.97	−207.13	134.34
	B-Alpha	0.99	−139.97	201.49
	B-Beta	0.99	−142.86	198.61
	Control	0.95	−214.81	126.66
B-Alpha	A-Alpha	0.80	−237.89	103.58
	A-Beta	0.99	−201.49	139.97
	B-Beta	1.00	−173.62	167.85
	Control	0.73	−245.57	95.90
B-Beta	A-Alpha	0.83	−235.00	106.46
	A-Beta	0.99	−198.61	142.86
	B-Alpha	1.00	−167.85	173.62
	Control	0.76	−242.68	98.78
Control	A-Alpha	1.00	−163.05	178.41
	A-Beta	0.95	−126.66	214.80
	B-Alpha	0.73	−95.90	245.57
	B-Beta	0.76	−98.78	242.68

A: traditional access cavity, B: contracted access cavity, Alpha: microhybrid composite, and Beta: self-adhesive composite.

**Table 4:** Spearman correlation test between fracture resistance test and cervical interfacial sealing ability, and significance level.

			Fracture Resistance	Sealing Ability
Spearman’s rho	Fracture resistance	Correlation Coefficient	1.00	0.226
		Sig. (2-tailed)	.	0.123
		*n*	60	48
	Sealing Ability	Correlation Coefficient	0.226	1.00
		Sig. (2-tailed)	0.123	.
		*n*	48	48

## Data Availability

The raw data supporting the conclusions of this article will be made available by the authors on request.
